# Exoskeleton home and community use in people with complete spinal cord injury

**DOI:** 10.1038/s41598-020-72397-6

**Published:** 2020-09-24

**Authors:** Rosanne B. van Dijsseldonk, Ilse J. W. van Nes, Alexander C. H. Geurts, Noël L. W. Keijsers

**Affiliations:** 1 Department of Rehabilitation, Donders Institute for Brain, Cognition and Behaviour, Radboud University Medical Center, Nijmegen, The Netherlands; 2 Department of Research, Sint Maartenskliniek, Nijmegen, The Netherlands; 3 Department of Rehabilitation, Sint Maartenskliniek, Nijmegen, The Netherlands

**Keywords:** Spinal cord diseases, Clinical trials, Rehabilitation

## Abstract

A consequence of a complete spinal cord injury (SCI) is the loss of gait capacity. Wearable exoskeletons for the lower extremity enable household and community ambulation in people with SCI. This study assessed the amount, purpose, and location of exoskeleton use in the home and community environment, without any restrictions. The number of steps taken was read from the exoskeleton software. Participants kept a daily logbook, and completed two user experience questionnaires (Quebec User Evaluation of Satisfaction with assistive Technology (D-QUEST) and System Usability Scale (SUS)). Fourteen people with a complete SCI used the ReWalk exoskeleton a median of 9 (range [1–15]) out of 16 ([12–21]) days, in which participants took a median of 3,226 ([330–28,882]) steps. The exoskeleton was mostly used for exercise purposes (74%) and social interaction (20%). The main location of use was outdoors (48%). Overall, participants were satisfied with the exoskeleton (D-QUEST 3.7 ± 0.4) and its usability (SUS 72.5 [52.5–95.0]). Participants with complete SCI report satisfaction with the exoskeleton for exercise and social interaction in the home and community, but report limitations as an assistive device during daily life.

## Introduction

The incidence of spinal cord injury (SCI) is approximately 180,000 cases per year worldwide^1^. Half of the people with SCI have a complete lesion^2^, which has a huge impact on their daily lives^3^. For mobility, a manual wheelchair is the most common option. Over the last decade, an alternative locomotion option for people with SCI was introduced: a wearable exoskeleton. With an external active orthosis and supported by two crutches, the basic motions for ambulation (i.e., standing-up, sitting-down, standing, and walking) can be initiated and controlled.

Until now, exoskeletons are mostly used in clinical settings, which has been shown to be feasible and safe for a broad range of patients with SCI^4,5^. As a gait retraining device, the exoskeleton is used to improve ambulatory capacity in patients with some residual leg motor function or in those for whom functional recovery is still possible (i.e., incomplete SCI). The potential of an exoskeleton for gait restoration was demonstrated by improving unassisted gait speed and walking distance after exoskeleton training^6^. Furthermore, two non-walkers with an incomplete SCI became walkers after exoskeleton training^4,7^. Hence, for people with incomplete SCI, ‘therapeutic’ exoskeleton use has the potential to improve ambulatory capacity independent of the exoskeleton.

In patients without (the potential for) ambulatory capacity (i.e., complete SCI), who are wheelchair users for their mobility, an exoskeleton can be used as an assistive device in order to walk. However, exoskeleton use as an assistive device without physical assistance from a trainer requires an intensive training period^7–9^. In our previous study, we have shown that more than half of the participants with a complete SCI could use an exoskeleton independent of assistance under supervision of a buddy after 24 training sessions^9^. Moreover, most of these participants were able to perform multiple advanced skills during independent walking (e.g. walking up and over a sloping doorstep) and standing (e.g. moving a cone at chest height)^9,10^, which strengthens the idea of exoskeleton home use during some daily activities. Another advantage of exoskeleton use are the potential health benefits^11,12^. Because of the possibility to stand and walk in an upright position, exoskeletons may help to prevent secondary health complications, such as spasticity^11,12^, impaired bowel function^13^, and related loss of quality of life^11^. From this perspective, exoskeletons are used as an exercise device to promote physical health and well-being by reducing secondary health complications. However, to actually reduce secondary health complications, the frequency and intensity of exoskeleton use are critical. The intensity of walking with an exoskeleton is similar to regular physical activities performed at a moderate intensity^14,15^, which is known to yield health benefits^12^. In contrast to the use of conventional knee–ankle–foot–orthoses, the use of exoskeletons is not physically exhausting^16,17^, which is why they can be used more regularly^14^. This strengthens the idea of continued exoskeleton use at home and/or in the community^18^.

To date, one study described a gym-based setting for exoskeleton use^19^. To our knowledge, no other studies have been described for the use of exoskeletons outside the clinical setting. Instead of in the actual home and community environment, exoskeleton use was investigated in a gym-based setting. Four participants with SCI were interviewed and reported that gym-based exoskeleton use had a positive impact on their lives and enhanced their perceived wellbeing and sense of community integration^19^. Yet, daily life entails much more than just the usage in a gym-based setting. According to Fritz et al.^20^, home and community use entails engaging in age normative and meaningful activities such as meeting friends at a pub, attending a graduation ceremony, performing one’s job, or going on holiday^20^. In addition to these social activities, home and community use also entails household chores such as cooking or doing the laundry. The question thus remains whether exoskeletons are already applicable in these settings^21^. Up to now, the applicability and effectiveness of exoskeletons in the community have not been demonstrated. Some expected challenges for community exoskeleton use are the limited gait speed, the heavy weight during transport, and the need of a buddy^18,22^. Only when users had an exoskeleton at their disposal in the community, the full range of problematic scenarios and safety concerns become apparent^20^. Such information is an important step for further exoskeleton development. Hence, the main objective of the present study was to assess the amount, purpose, and location of exoskeleton use in the home and community environment by people with complete SCI. Users’ experiences and health related effects during this period were studied as well.

## Methods

### Participants

People with complete SCI who gained knowledge about the existence and availability of an exoskeleton through the media and who were interested in testing the potential of such an exoskeleton contacted the rehabilitation center of the Sint Maartenskliniek to participate in this study. The eligibility criteria have been described previously^10^. Adults in the chronic phase (> 6 months) after a motor complete SCI (American Spinal Injury Association Impairment Scale (AIS) A or B) between the levels Thoracic 1 (Th1) and Lumbar 1 (L1) were eligible. Persons with physical characteristics that would hamper proper functioning of the exoskeleton, such as severe spasticity (Modified Ashworth Scale > 3), body height more than 1.90 m or less than 1.60 m, body weight above 100 kg, or restricted range of motion at any hip, knee or ankle joint were excluded. Other exclusion criteria were inability to control crutches, inability to make a transfer from a regular chair to a wheelchair without the use of external support, and any co-morbidity or condition that could interfere with motor learning (e.g. stroke). Subjects with an increased risk of adverse events, such as those with osteoporosis, fractures of the lower extremities during the previous 2 years, balance disorders, neurogenic heterotopic ossification, or pregnancy, were also excluded. The increased risk of adverse events was checked by the rehabilitation physician through questions. From participant 7 onwards, osteoporosis was tested with a dual energy X-ray absorptiometry (DEXA)-scan at the hip. Before participants were allowed to use the exoskeleton at home or in the community, the following criteria had to be met:

1. Participants had to complete an 8-week exoskeleton training and achieve a skill level for safe home and community use (i.e. at least 17 final skills in the previously described Final-skills-test^10^).
2. Participants were required to have a buddy who received instructions about guiding the participant during multiple skills, including donning and doffing, sit-to-stand, and walking. In addition, a device related error was simulated so that both the participant and the buddy practiced trouble shooting via a graceful collapse (see van Herpen et al. for a more detailed description of the trouble shooting protocol^23^). This protocol was added to the current study from participant 7 onward, after the occurrence of a bone fracture as described in two case reports^23^.

### Equipment

The ReWalk Personal 6.0, a wearable robotic exoskeleton from ReWalk Robotics that enables powered hip and knee motion, was used. The exoskeleton provided user-initiated mobility through the integration of a wearable brace support, a computer-based control system, and motion sensors. The ReWalk Personal 6.0 has a Class II FDA clearance for use both in a clinical setting and in the home and community environment^24^.

### Protocol

**Figure 1:**
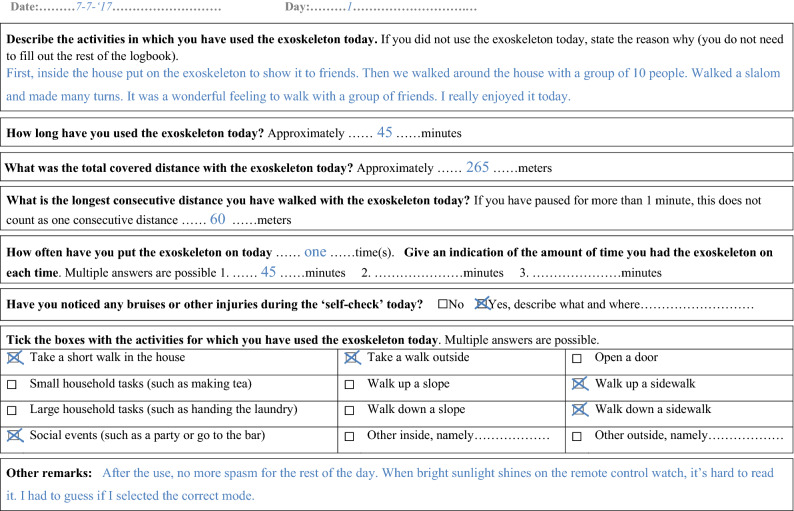
Example of the logbook during the period of home and community use.

Participants had no restrictions regarding the amount, purpose, or at which location they used the exoskeleton. For safety reasons, they were instructed not to use the exoskeleton without supervision of their buddy. The period of home and community use was at least two weeks with a maximum of three weeks per participant. The return date was agreed in advance, depending on the schedules of both the participant and the physical therapist. Before and after a period of home and community use, the number of steps taken was read from the exoskeleton software and notated. During the period of home and community use, participants filled in a logbook each day. If participants did not use the exoskeleton on a specific day, a short reason was given, while the other sections of the logbook were left blank. If participants did use the exoskeleton, the complete logbook was filled in (see Fig. 1). The logbook included questions regarding the performed activities with the exoskeleton, the amount of use, and skin integrity. Comments related to the overall experience with the exoskeleton and/or health related effects could be registered in the blank sections of the logbook. After the first week, the primary researcher (RD) called the participants by phone to check whether the logbook was filled in correctly and to monitor if they had experienced any problems. When the exoskeleton was returned to the clinic, the logbook was handed in and two user experience questionnaires were completed by the participants: (1) the Dutch version of the Quebec User Evaluation of Satisfaction with assistive Technology scale (D-QUEST 1–5 scale)^25^ and (2) the System Usability Scale (SUS 0–100 scale)^26^.

### Outcome measures

#### Exoskeleton use

Exoskeleton home and community use was assessed as (1) the amount of use, (2) the purpose of use, and (3) the location of use. The primary outcomes for exoskeleton use were the total number of steps taken, total number of days of usage, and total number of sessions. The total number of steps was extracted from the exoskeleton software (i.e. difference in total number of steps between the start and the end of the period of home and community use). The total days of usage and total number of sessions were obtained from the logbook (i.e. a completed page in the logbook indicated that they used the exoskeleton that day and multiple sessions on a day were written down). In addition, the amount of use per day (i.e. minutes of active time, total distance covered, and maximal distance covered without rest) was estimated by the participants and extracted from the logbook. Per session, the purpose and location of use were derived from the logbook and divided into various categories for the analysis. The five categories for *purpose* of use were: (1) individual exercise, (2) participation/social event, (3) exercise and social event, (4) explore indoor usability, and (5) other. The definitions of these purpose categories are described in Table 1. *Location* of use was divided into four categories: (1) outdoor use, (2) in home use, (3) indoor use at location (e.g. local fitness hall or indoor parking lot) and (4) mixture of use at the same day (i.e. indoor/home and outdoor). The percentage per category was calculated as the number of session per category divided by the total number of sessions. Reasons for non-use were extracted from the logbook.

**Table 1:** Definitions of the categories for purpose of exoskeleton use.

	Total distance walked		Presence of other people than the buddy	
	< 100 m	≥ 100 m	Yes	No
Individual exercise		X		X
Participation/social event	X		X	
Exercise and social event		X	X	
Explore indoor usability^a^	X			X
Other				

^a^Location is at home.

#### Exoskeleton experience

The exoskeleton experience was assessed as the satisfaction, importance, and usability of different aspects of the exoskeleton. In addition, other experiences with the exoskeleton (e.g. fall or device error) that were recorded in the logbook were evaluated. Satisfaction was derived from the 12-item D-QUEST questionnaire^25^ and divided into satisfaction with the assistive device (8 items), satisfaction with the service (4 items), and overall satisfaction (average). The satisfaction (sub)scores ranged between 1 and 5, with a higher score indicating greater satisfaction. Item analysis of satisfaction scores was performed, in which an item score of 1, 2 or 3 was considered dissatisfied^27^. In addition, the importance of each item was analysed by calculating how frequent a particular item was indicated by the participants as one of the three most important items. Usability was derived from the 10-item SUS questionnaire^26^ and evaluated as the overall usability and at item level. Each SUS item was scored from 0 (low usability) to 4 (high usability). The overall SUS score was calculated by multiplying the sum of the item scores by 2.5 and ranged between 0 and 100^26^, with a higher score indicating better usability. A mean SUS item score below 3 was considered low usability.

#### Health related effects

If participants reported health related effects in the logbook, these effects were categorised into positive (e.g. less neuropathic pain or improved mental health) and negative (e.g. increased pain or spasticity) effects. All negative effects were analysed regarding whether they resulted in non-use of the exoskeleton.

### Statistical analysis

The distribution of the data was tested for normality using a Shapiro–Wilk test. All outcome measures were analysed with descriptive statistics (mean and standard deviations). In case the assumption of normality was violated, median and ranges were calculated. The distribution of the amount, purpose, and location of exoskeleton use was reported using percentages (for grouped results) and frequencies (for individual results). In addition, frequency analysis of the user experiences and health related effects were reported.

### Ethics approval and consent of participants

All participants gave written informed consent in accordance with the Declaration of Helsinki. All research activities were carried out in accordance with the guidelines and regulations of the Medical Research involving Human Subjects Act (WMO) and the Netherlands Code of Conduct for Research Integrity. The study was approved by the medical ethics committee of Arnhem-Nijmegen (2016-2418) and the internal review board of the Sint Maartenskliniek.

## Results

### Exoskeleton use

**Figure 2:**
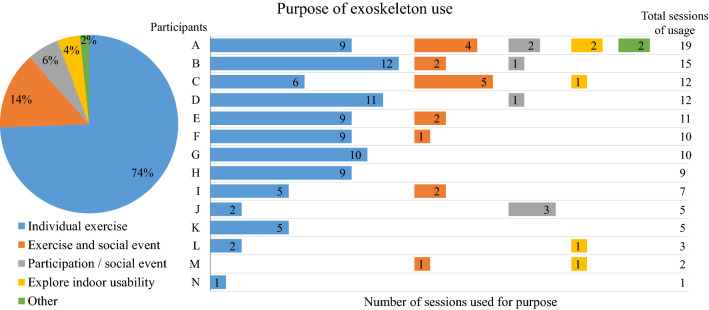
Purpose of exoskeleton use during the period of home and community use.

**Figure 3:**
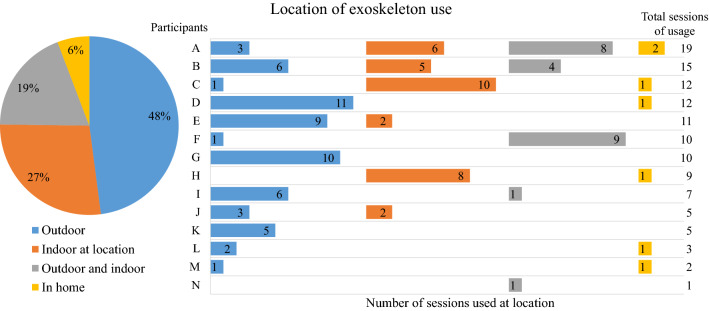
Location of exoskeleton use during the period of home and community use.

Fourteen participants had the exoskeleton at their disposal for home and community use. An overview of the participant characteristics is given in Table 2. Median exoskeleton use was 9 (range 1–15) out of 16 (range 12–21) days, in which participants performed a median of 9.5 (range 1–19) sessions and took a median of 3,226 (range 330–28,882) steps. An overview of the amount of exoskeleton use per participant is given in Table 3. Per day, the estimated median active time was 46 (range 19–84) minutes, during which the median estimated total distance covered was 243 (range 22–1,367) meters and the median estimated maximal distance covered without rest was 120 (range 12–1,125) meters (Table 3). The subject-reported primary purpose of exoskeleton use was for individual exercise (90 out of 121 sessions, 74% of all sessions) (Fig. 2). Other purposes of exoskeleton use were a combination of exercise and social event (14%, 17 sessions), participation/social event (6%, 7 sessions), explore indoor usability (4%, 5 sessions), or other (2%, 2 sessions). The location of exoskeleton use was mostly outdoors (58 out of 121 sessions, 48% of all sessions) (Fig. 3). The remaining location of exoskeleton use was 27% (33 sessions) indoors (e.g. gym or indoor parking lot), 19% (23 sessions) combination of indoors and outdoors, and 6% (7 sessions) at home. The following factors were mentioned as reasons for non-use of the exoskeleton: weather conditions (i.e. storm (participant I) and snowstorm (participant M)), becoming a father (participant J), dependence on buddy availability (participant K), and not meeting expectations (participant L).

**Table 2:** Participant characteristics.

	Total (N = 14)
Sex (male/female)	7/7
Age (years), median [min–max]	29 [24–49]
Time post injury (years), median [min–max]	6.25 [0.75–27]
Neurological level of SCI (thoracic), median [min–max]	Th9 [Th4–L1]
Classification of SCI level (low (Th7–12)/high (Th1–6))	8/6
AIS* (A/B)	13/1

*AIS = American Spinal Injury Association Impairment Scale.

**Table 3:** Amount of exoskeleton use during the period of home and community use.

Participant	Number of days at home	Primary: total exoskeleton use			Secondary: estimated exoskeleton usage per day (average)		
		Days of usage (%)	Number of sessions	Number of steps	Active time (minutes)	Total distance covered (m)	Distance covered without rest (m)
A	20	15 (71%)	19	3,065	59	245	95
B	17	15 (88%)	15	11,562	50	296	162
C	18	12 (67%)	12	4,358	38	199	58
D	17	11 (65%)	12	7,044	79	318	80
E	18	9 (50%)	11	3,276	48	344	200
F	15	10 (67%)	10	28,882	81	1,367	1,125
G	15	10 (67%)	10	5,098	19	207	132
H	15	9 (60%)	9	3,184	53	258	120
I	15	7 (47%)	7	10,333	64	727	590
J	15	5 (33%)	5	367	21	22	12
K	17	5 (29%)	5	572	84	240	120
L	17	3 (18%)	3	655	42	183	45
M	15	2 (13%)	2	768	28	220	210
N	12	1 (8%)	1	330	40	150	75
Median [min–max]	16 [12–21]	9 (55%) [1–15] (8–88%)	9.5 [1–19]	3,230 [330–28,882]	49 [19–84]	243 [22–1,367]	120 [12–1,125]

### Exoskeleton experience

**Figure 4:**
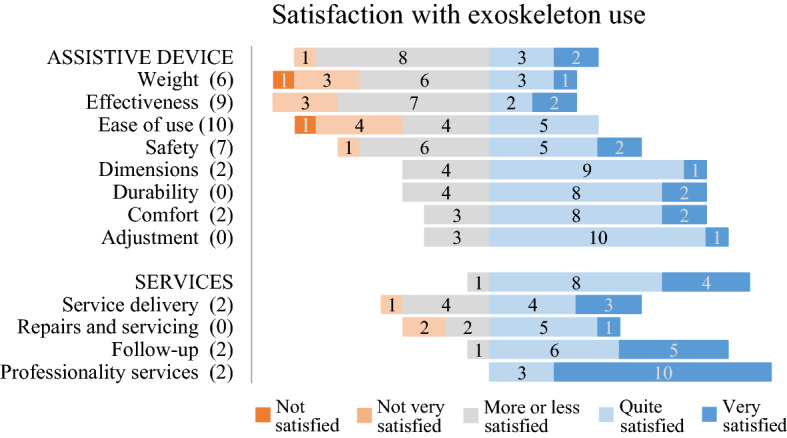
Distribution of the satisfaction scores on the items of the Quebec User Evaluation of Satisfaction with Assistive Technology (D-QUEST). A satisfaction score of ‘more or less satisfied’ or less (D-QUEST ≤ 3) was considered dissatisfied. The numbers in brackets represent how frequent an item was indicated within the top 3 most *important* items of the D-QUEST by the participants.

The average satisfaction with the exoskeleton was rated as 3.7 ± 0.4 (D-QUEST total), 3.5 ± 0.4 (subscale assistive device), and 4.2 ± 0.5 (subscale service). An overview of the item analysis of satisfaction and importance is shown in Fig. 4. ‘Weight’, ‘Effectiveness’, ‘Ease of use’, and ‘Safety’ were most frequently scored as dissatisfied (D-QUEST item score ≤ 3) and—at the same time—indicated as important. The usability of the exoskeleton was rated with a median of 72.5 [52.5–95.0]. Two SUS items had a mean SUS score below 3, indicating low usability (‘I needed to learn a lot of things before I could get going with this system (2.1 ± 1.5)’ and ‘I found the various functions in this system were well integrated (2.3 ± 0.8)’). Other experiences that participants recorded in the logbook were one fall and one device error (participant B). These incidences did not lead to health complications. Regarding the device error, the exoskeleton use was interrupted for three days until the error was resolved.

### Health related effects

Five patients reported positive health related effects in four domains: effects on social and mental health (n = 5), decreased spasticity (n = 3), reduced neuropathic pain (n = 1), and increased range of motion of the hip and back (n = 1). Five participants reported negative health related effects, among which muscle or joint pain (n = 4), skin damage (n = 2), increased spasticity (n = 1), and fecal incontinence problems (n = 1). In two participants who reported shoulder pain, exoskeleton use was stopped for one day (participant H) and during the remaining period (participant N), respectively.

## Discussion

This study assessed the amount, purpose, and location of exoskeleton use in the home and community environment in people in the chronic phase of motor complete SCI. In addition, users’ experiences and health related effects during the home and community use were studied. Fourteen people with a complete SCI used the exoskeleton 9 out of 16 days. The exoskeleton was mostly used for exercise purposes and social interaction. In half of the sessions the location of use was outdoors. Overall, participants were satisfied with the exoskeleton and its usability.

There was a large variation in the amount of exoskeleton use between the participants. The days of usage ranged from 1 to 15, during which 330 to 28,882 steps were taken. The low amount of use in some participants could be attributed to exoskeleton related factors (i.e. shoulder complaints, not meeting expectations, and dependence on buddy availability) or external factors (i.e. snowstorm and becoming a father). In contrast, more than half of the participants used the exoskeleton at least one out of two days. Because of the novelty of the exoskeleton and the relatively short time period of exoskeleton availability, participants probably optimally exploited the possibilities. Thus, although our results are likely representive for short term home and community use, they may be an overestimation with regard to long term exoskeleton use.

Based on the purpose and location of use, the potential of a wearable exoskeleton can be identified. The expected potential of an exoskeleton has previously been addressed in a qualitative study by Manns and colleagues^28^. They found that some participants expected the best potential for outdoor exoskeleton ambulation, whereas others expected better potential for functional indoor use during daily life activities (e.g. cooking while standing)^28^. Yet, only when participants have an exoskeleton at their disposal in the home and community setting, the full potential of an exoskeleton can be truly assessed. In the current study, all participants used the exoskeleton for exercise purposes and two thirds of the participants used it for social interaction, either with or without exercise. For exercise and social interaction purposes, the exoskeleton was used at locations with large, open, and smooth surfaces, either outdoors or indoors. In contrast, the exoskeleton was barely used at home. Indoor usability at home was explored by only four participants (and repeated once by merely one participant). The current findings support the general thought that an exoskeleton has a high potential to be used as an exercise device and for social interaction at eye-level, but a low potential as an assistive device for supporting daily activities^18,20^.

Despite the shortcomings of the investigated exoskeleton as an assistive device during daily life activities, participants were generally satisfied with its use. Based on conversations with the participants, we assume that participants evaluated their overall satisfaction of the exoskeleton with respect to the current use and their expected applications. Most participants used it as an exercise device and for social interactions and indicated the following aspects of improvement in order of importance: ease of use, effectiveness, safety, and weight. Ease of use has also been rated as most important for manual wheelchair^29,30^ and walker use^29^. One difference between wheelchair / walker use and exoskeleton use is, the requirement of a buddy during exoskeleton use, thus potentially limiting the individual’s independence. Ten of the participants reported that the buddy was a hindrance, as has been reported in other studies^18,22^. The buddy requirement presumably influenced the other three aspects of improvement (effectiveness, safety, and weight) as well. The comments related to ‘satisfaction with effectiveness’ (e.g. the degree to which the device meets one’s needs) reflected participants’ desire for independent functional use. The participants also commented that they felt most safe with the buddy constantly guarding them during use and that because of the weight (~ 27 kg) of the device, a buddy was needed for transporting the device in and out of their cars. Since detailed information regarding transportation was not investigated in the current study, future studies should investigate the implications and issues regarding transport. Nevertheless, participants also emphasized that there are no other devices that enable similar options for exercise or social interaction in an upright position. Therefore, the overall satisfaction with the exoskeleton and its usability was considered as good, although less need for a buddy and improved transportability is desired. For these reasons, it is important to investigate how the exoskeleton can be further improved with respect to the applications that users have in mind.

For application at home and in the community, two exoskeletons are currently FDA and CE approved (ReWalk and Indego^24,31^). The main difference between these exoskeletons is that the Indego exoskeleton is modular and weighs less^31^, which facilitates transportation. Despite this difference in modularity, both exoskeletons require the use of upper extremity support (i.e. crutches) for balance, are only allowed to be used with a trained buddy, and have the same three options for mobility (sit, stand, and walk^24,31^). Although in the current study the ReWalk exoskeleton was investigated, we expect similar findings for other currently commercially available exoskeletons that rely on upper extremity points of contact with crutches for balance.

In the current study, participants with a low (Th7–12, n = 8) and high (Th1–6, n = 6) complete SCI who could control crutches were included, reflecting a large range of the complete SCI population. However, selection bias is likely, because only people with a complete SCI who were interested in exoskeleton use and who could commit to the exoskeleton training protocol participated. Furthermore, only participants who achieved a skill level for safe home and community use (i.e. at least 17 final skills in the previously described Final-skills-test^10^) were included. Therefore, the results should be interpreted with caution and are not generalizable to the whole SCI population.

In conclusion, the exoskeleton investigated in this study (ReWalk) has a good potential to be used as an exercise device in the home and community environment. For this purpose, participants with a motor complete SCI were satisfied with its use, but wished for technological improvements to reduce the need for a buddy and to improve the overall transportability of the device. In addition, the exoskeleton enables social interaction at eye-level. Therefore, the use of an exoskeleton has the potential to contribute to the physical and mental health in people with complete SCI. As an assistive device during daily life activities, the exoskeleton has many limitations. Further exoskeleton development should, thus, look at other (more functional) applications that are important to their potential users.

## Data Availability

All data associated with this study are present in the paper.
